# HIV-1 Accessory Protein Vpr: Relevance in the pathogenesis of HIV and potential for therapeutic intervention

**DOI:** 10.1186/1742-4690-8-25

**Published:** 2011-04-13

**Authors:** Michael Kogan, Jay Rappaport

**Affiliations:** 1 Department of Neuroscience, Department of Neuroscience, Center for Neurovirology, Temple University School of Medicine, 3500 North Broad Street, Philadelphia, PA 19140, USA

## Abstract

The HIV protein, Vpr, is a multifunctional accessory protein critical for efficient viral infection of target CD4^+ ^T cells and macrophages. Vpr is incorporated into virions and functions to transport the preintegration complex into the nucleus where the process of viral integration into the host genome is completed. This action is particularly important in macrophages, which as a result of their terminal differentiation and non-proliferative status, would be otherwise more refractory to HIV infection. Vpr has several other critical functions including activation of HIV-1 LTR transcription, cell-cycle arrest due to DCAF-1 binding, and both direct and indirect contributions to T-cell dysfunction. The interactions of Vpr with molecular pathways in the context of macrophages, on the other hand, support accumulation of a persistent reservoir of HIV infection in cells of the myeloid lineage. The role of Vpr in the virus life cycle, as well as its effects on immune cells, appears to play an important role in the immune pathogenesis of AIDS and the development of HIV induced end-organ disease. In view of the pivotal functions of Vpr in virus infection, replication, and persistence of infection, this protein represents an attractive target for therapeutic intervention.

## Introduction

**Figure 1:**
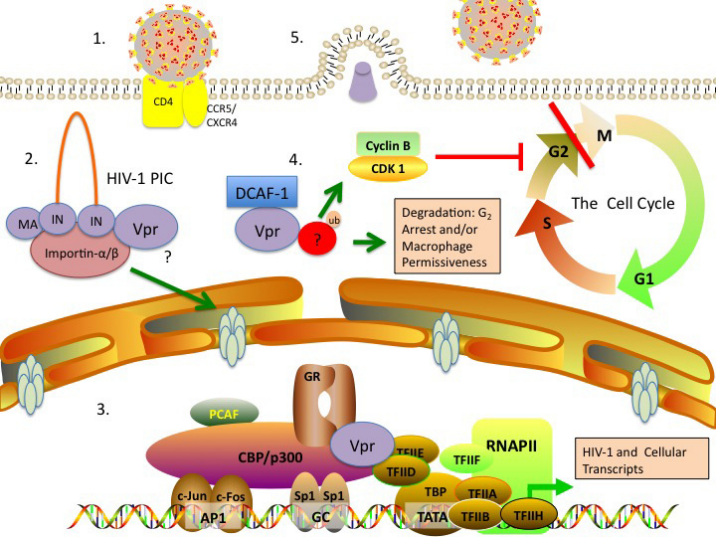
**The role of Vpr in HIV-1 infection and host permissiveness**. 1). HIV-1 enters human cells via interaction with cell-surface receptors CD4 and co-receptors CXCR4 (T-cell tropic viruses) or CCR5 (macrophage tropic viruses). The virus fuses with the cell surface membrane introducing genetic material and virion proteins, which include gag proteins that comprise the matrix and nucleocapsid, the latter containing significant quantities of Vpr. 2). Vpr promotes the binding of the PIC (including MA, integrase (IN) and proviral DNA) to importins and nucleoporins, thereby facilitating nuclear entry of HIV-1 provirus into the nucleus of non-dividing cells. 3). Vpr binds to the p300/transcription factor initiation complex. This binding activity may recruit additional elements to the promoter, such as glucocorticoid receptor (GR). Alternatively, Vpr may bind to GR bound to GRE elements in the promoter to recruit the p300/TF complex. This results in both increased HIV-1 production, and the regulation of cellular genes that may increase viral permissiveness. 4). Vpr induces G_2 _cell-cycle arrest by promoting phosphorylation of Chk1, which increases viral production. Interestingly, the biochemical properties that contribute to this effect may be important in HIV-1 production in cells that do not divide. This property is dependent on the degradation of an unknown factor, which is recruited to Vpr via DCAF-1 interaction. The factor(s) involved in G_2 _arrest and viral permissiveness may be overlapping or unique. 5). HIV-1 buds from the cell, promoting further infection and pathogenesis.

Human immunodeficiency virus type 1 (HIV-1) is a lentiviral family member that encodes retroviral Gag, Pol, and Env proteins along with six additional accessory proteins, Tat, Rev, Vpu, Vif, Nef, and Vpr. Viral protein R (Vpr) is a 96 amino acid, 14 kDa protein that was originally isolated almost two decades ago [1,2] and is highly conserved in both HIV-1 and simian immunodeficiency virus (SIV) [3-5]. Numerous investigations over the last 20 years have shown that Vpr is multifunctional. Vpr mediates many processes that aid HIV-1 infection, evasion of the immune system, and persistence in the host, thus contributing to the morbidity and mortality of acquired immunodeficiency syndrome (AIDS). Vpr molecular functions include nuclear import of viral pre-integration complex (PIC), induction of G_2 _cell cycle arrest, modulation of T-cell apoptosis, transcriptional coactivation of viral and host genes, and regulation of nuclear factor kappa B (NF-κB) activity. The numerous functions of Vpr in the viral life cycle suggest that Vpr would be an attractive target for therapeutic intervention. A summary of the effects of Vpr on HIV-1 infectivity and permissivness is provided in Figure 1.

## Vpr mediates nuclear transport of the HIV-1 pre-integration complex and enables macrophage infection

In non-dividing mammalian cells, free diffusion of cellular contents into the nucleus is limited to components that are less than 40 kDa [6]. Retroviruses require entry into the nucleus to replicate and are, therefore, naturally restricted to those cells that undergo mitosis. Lentiviruses such as HIV-1, however, are unique among retroviruses in that they able to infect non-dividing cells [7,8]. Early studies have shown that the HIV-1 PIC can enter the nucleus by an active process without causing structural damage to the nuclear envelope [9,10]. Indeed, Vpr has been found to localize to the nucleus when expressed alone or in the context of viral infection [11-13]. Furthermore, Vpr has been demonstrated to play an important role in the localization of the HIV-1 PIC to the nucleus and a critical role in the infection of non-dividing cells, as discussed in more detail later in this review. The role of Vpr in the nuclear import of the PIC is illustrated in Figure 1. The PIC is targeted to the nucleus by Vpr via interaction with importin-α, ultimately promoting binding to nuclear pore proteins.

In addition to Vpr, viral proteins matrix antigen (MA) and integrase (IN), have been shown to participate in nuclear entry. MA and IN both have a functional nuclear localization sequence (NLS) and the nuclear import function of these proteins requires the action of cellular partners importin-α and -β. Interestingly, it was reported that IN can be sufficient for import of PICs when over expressed in the absence of Vpr or MA [14]. Furthermore, the HIV-1 central DNA flap and capsid protein (CA) have also been reported to play a role in PIC nuclear targeting [15,16]. Unlike Vpr, these components appear to promote nuclear localization by a linked mechanism involving the uncoating of the PIC. It appears that there are multiple and sometimes redundant nuclear localization signals involved in nuclear entry of the HIV PIC. Two classical pathways have been characterized for the transport of proteins across the nuclear pore complex (NPC): the NLS and M9-dependent pathways (for review see [17]). The former pathway involves the binding of NLS signal containing peptide to importin-α via central armadillo repetitive motifs. Importin-α binds to importin-β via an amino-terminal importin-β-binding (IBB) domain [18,19]. The binding of the classical NLS to importin-α is not possible until this IBB binding to importin-β occurs, which causes importin-α to expose an internal NLS [19]. This multi-protein structure then interacts with the NPC at which point importin-β transports this NLS component into the nucleoplasm.

Two other proteins, GTPase Ran/TC4 and NTF2, are also involved in NLS mediated transport [20-24]. Importin-α serves as an adaptor molecule by bridging NLS containing compounds to nuclear transport machinery. It has been reported, however, that importin-α can facilitate nuclear entry of Ca^2+^/calmodulin-dependent protein kinase type IV (CaMKIV) without importin-β [25]. Further, importin-β can transport cyclin B1/Cdc2 without Ran, suggesting that mechanisms of import exist that can utilize one or both importins [26]. In the M9-dependent pathway, transportin facilitates both nuclear import and export of RNA binding protein hnRNP A1 by recognizing an M9 signal sequence [27-31]. M9 mediated nuclear trafficking also depends on the function of Ran/TC4, just as in the classical NLS system [32].

Vpr nuclear localization seems to utilize cellular machinery in a unique way that is independent of the classical NLS and M9 pathways. While viral MA is inhibited by NLS blocking peptides and dominant-negative importin-α (residues 244-529), Vpr nuclear entry is not affected by either treatment strongly supporting the notion that Vpr functions in an NLS-independent manner [14]. Vpr mediated import is also unaffected by treatment with RanQ69L, a dominant-negative form of Ran, that inhibits both M9 and NLS pathways [32-34]. GTPγS, a nonhydrolyzable GTP that inhibits Ran function [23,35,36], has no effect on Vpr localization, further suggesting that Vpr localizes in a non-conventional, Ran-independent manner [37]. Vpr mediated karyophilic activity is starkly contrasted to that of classical SV40 NLS, which requires the presence of importin-α/β and Ran GTP[38]. Further, Vpr nuclear localization appears to be independent of energy, or at least requires less energy than conventional transport. Addition of adenosine triphosphate (ATP) or treatment with apyrase, which lowers NTP levels, affected the localization of classical NLS bearing proteins but had no effect on Vpr localization [34,37]. Another study suggested that Vpr can enter the nucleus via two different mechanisms; one involving importin-α and another involving energy [39]. In summary, Vpr may use importin-α in a non-conventional, energy independent manner, but may also use a yet undetermined mediator in the absence of importin-α in a process requiring ATP.

In accord with Vpr's ability to promote nuclear localization of the PIC, Vpr has been shown to be essential for productive HIV-1 and HIV-2 infection of macrophages [40-43]. While HIV-1 IN can compensate for loss of Vpr at high MOI of HIV-1 [14,44], other studies suggest that Vpr deficient HIV-1 is non-productive in macrophages at least partly due to the inability to penetrate nuclei of non-dividing mononuclear cells [38,41,45-50]. Further, it was shown that Vpr is directly involved in targeting the HIV-1 PIC to the nuclear envelope [51]. It appears that mucosal infection of HIV-1 involves the transmission of likely a single virus per patient, as determined by sequence analysis of founder virus [52]. This claim from initial studies has been greatly strengthened by a recent study following patients early during acute infection and the analysis of HIV specific escape epitopes variants by deep sequencing [53]. Therefore, as the multiplicity of infection during transmission is quite low, it would be expected that Vpr would be required during this event. Later in infection, when viremia is elevated, IN and MA may have appreciable effects on PIC entry, although this remains to be proven. Interestingly, it was also reported that Vpr's nuclear localization and consequent G_2 _arrest properties are important in HIV-1 infection of primary CD4^+ ^T-cells irrespective of proliferative status [54](reviewed in: [55]). HIV-1 clearly infects resting T-cells *in vivo*, where Vpr mediated transport of the PIC into the nucleus would be expected to have importance. The action of Vpr, however, appears to be required for CD4^+ ^T-cell infection, even under conditions promoting proliferation (i.e. in the presence anti-CD3 and IL-2 treatment [54]). It is likely, therefore, that the transport of the PIC across the nuclear envelope is important in both T-cells and macrophages *in vivo*.

In addition to Vpr, there are other requirements for viral replication in non-dividing cells. The viral capsid protein, CA, appears to support this role in that mutations in CA disrupt the cell cycle independence of HIV-1 infection [56]. The role of CA appears to be independent of nuclear import as one of the mutants in CA exhibited a defect in replication in non-dividing cells beyond the nuclear entry point. The necessity of Vpr's karyophilic properties for the infection of actively dividing cells suggests that the targeting of the PIC to the NPC is a generally required aspect of lentiviral infection, regardless of cell cycle progression. In an evolutionary context, this may imply that lentiviruses evolved to infect non-dividing macrophages and expanded later to T-cells while retaining the use of already evolved infection machinery from the original, non-dividing, target cell population. Indeed, macrophages are a common target of all known naturally occurring lentiviruses [57]. Furthermore, T-cell infection is common only to lentiviruses that cause immunodeficiency, further suggesting that these cells were later targets of tropism during lentivirus evolution. In this model, Vpr may contribute to nuclear localization in general, whereas other components, such as CA, may facilitate additional processes necessary for productive infection of cell cycle arrested cells. In conclusion, Vpr seems to be an important mediator of human lentiviral infection, at least in part due to nuclear localization properties. This effect may be most important during periods of low HIV-1 plasma viremia or transmission from person to person.

## Correlations between Vpr's structure and nuclear localization function

Structural studies have been invaluable to understanding HIV-1 viral interaction with host cells, including non-dividing macrophages. Relatively recent structural studies have identified three alpha helical domains, α-H1 (13-33), α-H2 (38-50), and α-H3 (55-77) as well as other structural features capable of mediating diverse biological functions [58]. Indeed, Vpr's structure allows for direct binding to many cellular proteins, which likely enables Vpr to mediate functions such as nuclear import and G_2 _arrest. All three alpha helices have been implicated in Vpr mediated nuclear localization [12,13,59-62], while the G_2 _arrest property has been attributed mainly to the C-terminal region of Vpr [59]. However, as the nuclear import, promoter transactivation, and G_2 _arrest properties of Vpr seem to not only be related, at least on a structural level, they also may be jointly attributed to specific physiological properties of Vpr in productive HIV-1 infection of macrophages [63].

Vpr mediates nuclear localization by binding to importin-α via residues located within the alpha helices. While some studies initially reported a low affinity of Vpr for importin-α [37], others have found that Vpr binds to importin-α using other techniques [50,51,64]. Vpr/importin-α binding was shown to be non-competitive with that of the classical the NLS found on MA [65]. Kamata and others demonstrated that regions 17-34 (αH1) and 46-74 (αH2+αH3) can both independently localize to the nucleus, albeit to a lower extent than an identified bona fide Vpr NLS consisting of residues 17-74 [66]. Mutations in αH1, αLA (L20,22,23,26A), as well as in αH2+αH3, I60P and L69P, completely ablated the ability of the individual peptides to localize to the nucleus. Later, Kamata and others found that Vpr αH1 and αH3 both bind importin-α, that the IBB domain of importin-α primarily determines this interaction, and that the C-terminal domain of importin-α, 393-462, is necessary for nuclear localization of Vpr [39]. Although, an importin-α lacking an IBB still facilitated import of Vpr, a mutation in Vpr's first alpha helix, αLA, impaired importin-α binding and nuclear localization but still showed perinuclear accumulation. In contrast, a mutation in the third alpha helix, L67P, failed to localize to both the nuclear and perinuclear areas, but still permitted binding to importin-α. The authors concluded that binding to importin-α requires only the first alpha helix and that the third alpha helix serves to localize Vpr to the perinuclear area independently of importin binding. Previous findings from other investigators also showed that the use of IBB peptides failed to inhibit Vpr mediated nuclear localization. This suggests that importin-α may be essential for Vpr's karyophilic properties but that the direct interaction between importin-α and Vpr may not be essential [34]. Hitahara-Kasahara and others showed that importin-α1, α3, and α5 isoforms are all able to induce Vpr mediated nuclear import [38]. Importin-α was shown to be essential for HIV-1 replication in macrophages, suggesting that importin-α nuclear import is a vital process in the infection of these cells. Furthermore, a recent study found that Vpr does not bind to importin-α2 or importin-α2/β1 heterodimers, suggesting that cell-line specific expression of importins may regulate Vpr's karyophilic properties [46]. In summary, these studies suggest that importin-α is important for Vpr-mediated nuclear translocation, but the exact nature of this mechanism is still under investigation.

In addition to the reported binding interaction with importin-α, Vpr has been demonstrated to bind directly to nuclear pore proteins [47,49-51,67]. Vpr mutants F34I and H71R have been found to lose the ability to localize to perinuclear areas, suggesting that these residues are involved in nuclear pore interaction [50]. These mutants were still found in the nucleus, which is not surprising considering that Vpr is less than 40kDa. The F34I mutant showed lower binding to importin-α and Nsp1p, a member of the nuclear pore complex. WT Vpr colocalizes with importin-β and nuclear pores in perinuclear regions and binds both Pom 121 and very weakly to Nsp1p [47]. An A30P mutant lacked these abilities.

FXFG regions on nucleoporins, a form of phenylalanine-glycine (FG) repeat, have been reported to interact with cytoplasmic proteins involved in nuclear import [22,68,69]. Vpr was reported to bind to FXFG containing proteins p54 and p58 as well as to the FXFG region of Nup1 [51]. Further, addition of Vpr was shown to stabilize the binding of importin-α/β to Nup1 FXFG. Another report failed to show interaction between Vpr and FXFG of Pom121, but instead demonstrated that the alpha helices of Vpr interact with hCG1 by binding to a non-FG repeat region located in the N-terminal region on residues 49-170 [67]. This area has no known homology to binding motifs and has no known binding partners. In a later study, it was found that four Vpr mutants L23F, K27M, A30L, and F34I, which all occur on one face of the first alpha helix, have impaired hCG1 binding and fail to show nuclear localization [49]. Thus, it seems that Vpr is able to bind to importin-α as well as nucleoporin using the same residues on the first helix. In both cases, there is evidence that Vpr binding to nucleoporin components occurs in a way that is distinct from the classical NLS pathway.

The role of importin-β in the nuclear transport of Vpr is an aspect of the mechanism of Vpr's karyophilic properties that remains to be fully understood. Early studies showed that Vpr fails to bind importin-β [65] or that it binds at a low affinity [37]. Oddly, the latter study found greater affinity of Vpr to importin-β than to -α. Subsequent studies argued that Vpr's localization is importin-α, but not -β, dependent. Addition of importin-β to digitonin permeabilized cells, which was required for the classical SV40-NLS localization, was unnecessary for Vpr N17C74, a construct containing the minimal region for nuclear localization [38,66]. These studies also found that ΔIBB importin-α, which is unable to bind to importin-β, still caused nuclear translocation of N17C74. Previous studies demonstrating that the use of IBB peptides failed to inhibit Vpr localization also lend some support to these findings [34]. Further, importin-β siRNA failed to prevent N17C74 localization to the nucleus [38]. Vpr has also been shown to physiologically behave in ways similar to importin-β, leading some authors to suggest that Vpr replaces the role of importin-β, which, like Vpr, also binds to both importin-α and nuclear pores, in the nuclear translocation process [50]. Other studies, however, suggest that importin-β is necessary for Vpr's karyophilic properties. Papov and others found that Vpr prevents FXFG Nup 1 peptide mediated dissociation of MA with importin-α/β complexes and increases the affinity of importin-α to NLS [51,65]. Based on these findings Papov and others proposed that Vpr stabilizes the MA and IN NLS complex with importin-α/β to promote nuclear entry. A dominant negative form of importin-β, residues 71-876 [70] has also been shown to inhibit Vpr localization, further suggesting that importin-β plays a role in Vpr mediated nuclear targeting [34]. Recent studies have clearly shown binding of Vpr to importin-β3, but not to importin-β1 or to importin-α2/β1 complexes [46]. This may explain discrepancies in early findings that failed to find effects of isolated importin-β which were not necessarily applicable to other importin-β isoforms.

The respective roles of the alpha helices and the C-terminal region in nuclear localization and G_2 _arrest remain controversial. Through extensive mutational analysis, Mahalingam and others put forth a hypothesis that the nuclear localization function resides primarily in the alpha helices while the G_2 _arrest property is determined by the carboxyl-terminus [59]. Previous studies lend support to this assertion as the alpha helices, but not N-terminal or C-terminal regions were involved in nucleoporin binding by Vpr [67]. Other reports found that N17C74 Vpr, which lacks the C and N terminal regions and other Vpr constructs lacking the C-terminus are unimpaired in nuclear localization [11,66]. Although the C-terminal region closely resembles a classical NLS, this region does not have NLS function and Vpr functions independently of NLS binding [14,71]. Conversely, many other studies found that the C-terminal is necessary or sufficient for nuclear entry of Vpr [12,34,47,62,72]. The discrepancy between these studies remains unexplained. Interestingly, recent studies have shown that all three alpha helices are involved in Vpr oligomerization [63]. The authors reported that mutations that affected oligomerization did not prevent apoptosis induction by Vpr (a G_2 _arrest dependent property [73]). Nuclear localization, however, was perturbed for these mutants. These studies may suggest that karyophilic and cell cycle arrest properties rely on multiple domains that may be separable to some degree.

## Vpr functions as a coactivator of the HIV-1 long terminal repeat

While Vpr promotes infection of HIV-1 into non-dividing cells, the ability of Vpr to activate both viral and endogenous promoter activity likely contributes to increased viral replication and pathogenesis. Initially, it was observed that Vpr can reactivate cells latently infected with HIV-1 [74,75]. Later studies demonstrated more specifically that Vpr transactivates the HIV-1 long terminal repeat (LTR) as well as other promoters [76-78]. The U3 region of the HIV-1 LTR has several activating elements, which include NF-AT, glucocorticoid response elements (GRE), NRF, NF-κB, Sp1, a Tat responsive RNA element (TAR), and a TATA box [79-83]. Studies employing HIV-1 LTR indicator constructs demonstrated that Vpr acts via Sp1 sites [78]. Vpr binds to the Sp1/promoter complex and it has been proposed that Vpr exerts its effects by stabilizing promoter complexes containing multiple bound Sp1 proteins. Other studies, however, support the notion that Vpr transactivates primarily the -278 to -176 region of the LTR, which contains the GREs, while the NF-κB and Sp1 are utilized by Tat mediated transactivation [84].

Vpr appears to act as a coactivator in the presence of other activating elements but not on a bare promoter alone. Vpr was shown to bind transcription factor IIB (TFIIB), suggesting that the effect of Vpr is indeed due to coactivation rather than direct transcription factor function [76]. Vpr has also been demonstrated to potentiate the activation of the HIV-1 LTR by p300 [85] and was shown to form a complex with p300 and TFIIH to cooperatively induce GRE activation in a manner independent of G_2 _cell cycle arrest [86]. Consistent with these findings, a Vpr mutant deficient in p300 binding, I74,G75A, did not display this property. Several Vpr mutants including R73S, C76S, and Q21P have also been reported to lose HIV-1 LTR transactivation abilities [87]. Intriguingly, the R73S mutation imparted a dominant-negative phenotype with regard to transactivation. Vpr has also been reported to act cooperatively with Tat, another LTR coactivator. Their cooperative effect was disrupted by the Vpr R73S mutation [88]. Therefore, in the presence of Vpr, viral production is likely amplified via coactivation of the HIV-1 LTR by a mechanism that appears to be dependent on multiple binding sites within the viral LTR.

The glucocorticoid receptor (GR) has been a known target of Vpr function for more than a decade [89]. Originally, Vpr was shown to induce R-interacting protein 1 (Rip-1) nuclear translocation in a GR dependent manner and along with Rip-1 form a complex with GR. A later study showed that Vpr transactivates promoters containing GREs [90]. The authors also reported that Vpr L64A, a mutant for a signature GR binding motif LXXLL, was found to be defective for binding to GR and in GRE transactivation, but like WT Vpr, Vpr L64A retained the ability to bind TFIIB. A Vpr R80A mutant, which lacked G_2 _arrest, was unimpaired in GRE-mediated transactivation. This study also reported that Vpr/p300 synergy was amplified in the presence of dexamethasone. A later publication confirmed many of these observations for LXXLL Vpr mutants in the first and third alpha-helices, 22-26 and 64-68 respectively [91]. The authors reported that mutations in both helices were necessary to completely diminish GRE promoter activation. Subsequently, Kino and others identified Vpr mutants, F72, R73A and I74,G75A, which were unable to bind p300 and were therefore deficient in GRE transactivation [92]. Unlike Vpr L64A, these mutants were not reported to be transdominant, suggesting that Vpr L64A competes with WT Vpr for p300 binding. It is noteworthy that while some subsequent studies have found conflicting results [93], later research has solidified the notion that GR and Vpr function synergistically. Human Vpr interacting protein (hVIP/Mov34), which binds to both Vpr and GR, translocates to the nucleus following either dexamethasone or Vpr treatment, further suggesting that Vpr and GR form an functional complex within cells [94]. Vpr and GR also have a gain of function in inhibiting poly (ADP-ribose) polymerase 1 (PARP-1) nuclear translocation, which is a necessary event in NF-κB transcription [95]. It is worth noting that the effect of Vpr on NF-κB remains a controversial topic (discussed below in: "Vpr and immune dysfunction"). However, HIV-1 infection and NF-κB activation form a positive feedback loop [96,97], and Tat is known to induce the HIV-1 LTR synergistically with NF-κB [98], highlighting the importance of the NF-κB pathway for HIV-1 replication. Considering that NF-κB signaling is activated during HIV-1 infection, the role of Vpr in the context of HIV-1 infection may or may not be identical to studies using ectopic Vpr expression. In summary, these studies suggest that Vpr and GR function in a cooperative manner through a mechanism that involves direct binding, and this interaction is at least partly responsible for the transctivation of the HIV-1 LTR by Vpr. The interaction of Vpr with GR and elements of the LTR transcription complex, including p300 is illustrated in Figure 1.

Although Vpr appears to coactivate the HIV-1 promoter via GRE and generally behaves in a GR-dependent manner (with respect to transcriptional activation), the role of glucocortcoids on HIV-1 viral replication remains controversial. Several groups have reported altered hypothalamic-pituitary-adrenal (HPA) axis function in HIV-1 infection [99-104]. Additional *in vitro *molecular studies have reported that glucocorticoids suppress the HIV-1 LTR [105-109]. Kino and others reported that this effect depends on GR and is not influenced by Vpr [105]. These reports are seemingly in contradiction with aforementioned studies, which showed that Vpr transactivates the HIV-1 LTR and that Vpr enhancement of other promoter elements containing GREs is potentiated by glucocorticoids. Intriguingly, Laurence and others reported that the level of HIV-1 LTR activity in unstimulated cells is not diminished by dexamethasone, while phorbol ester induction of the HIV-1 LTR was attenuated by such treatment [106]. In contrast, some investigators have reported that glucocorticoids have an enhancing effect on HIV-1 LTR activity [110,111]. The latter study showed that this effect was seen only in the context of interleukin (IL)-6 and tumor necrosis factor alpha (TNF-α). Interestingly, a recent study found that extracellular Vpr was capable of increasing IL-6 production in an NF-κB and C/EBP-β dependent manner by stimulating Toll-like receptor 4 (TLR4) signaling in macrophages [112]. Glucocorticoids and TNF-α have also been shown to increase HIV-1 virus production [113]. Therefore, the effect of glucocorticoids on the HIV-1 promoter may be influenced by the presence or absence of pro-inflammatory signals. Increased levels of glucocorticoids have been associated with HIV-1 progression, although some reports suggest that these effects are due to immune system modulation rather than a direct effect on viral replication [12,114-116]. Subsequently, it was shown that RU486, a GR and progesterone receptor (PR) inhibitor, can reduce HIV-1 LTR activation by Vpr and attenuate virus production in X4 infected PBMCs as well as R5 infected macrophages [117]. In contrast, glucocorticoids can increase the permissiveness to infection of unstimulated PBMCs by HIV-1 [118]. These studies demonstrated that the viral life-cycle was blocked at a stage of infection before proviral integration. Interestingly, a similar block in HIV-1 replication was also shown to be abrogated by Vpr, further suggesting GR/Vpr cooperativity [41]. In summary, Vpr may have varying effects on the HIV-1 LTR depending on the context of proinflammatory and anti-inflammatory signals, in addition to GR pathways.

## The interrelationship of Vpr functions and their relevance to macrophage permissiveness and HIV-1 reservoirs

Numerous studies have focused on the role of Vpr in macrophage infection and permissiveness to HIV-1. However, the involvement of multiple properties of Vpr in these processes has made it difficult to exactly ascertain which features are most important for macrophage infection. Further, some studies have relied on mutation of individual residues to discern these effects. However, the mutants produced often show defects in multiple properties, which are clearly independent biologically, making the analysis of structural studies challenging. A confusing issue in the literature is that the "so called" G_2 _arrest function of Vpr, which is likely irrelevant to the status of terminally differentiated cells such as macrophages, has been associated in some studies with HIV-1 infectivity of such differentiated cells. Recent findings in the field, however, suggest the likelihood that both G_2 _arrest and another, yet unknown, cellular process use similar machinery and that the factors involved in these Vpr functions may have significant overlap.

Findings from mutational studies have suggested overlap in G_2 _arrest and localization of the HIV PIC to the nucleus. In a recent study the authors reported that the G_2 _arrest properties of Vpr depend on nuclear localization [49]. Jacquot and others showed that four Vpr mutations in the first alpha helix, Vpr L23F, K27M, A30L and F34I all exhibit both at least partially impaired G_2 _arrest and defective nuclear localization while Vpr mutants R80A and R90K were deficient in G_2 _arrest alone. While previous studies confirmed some of these results, they have also reported opposite results for the same mutations or support the notion that the two properties are independent [11,50,59]. It is noteworthy to mention that these two properties are completely separated in HIV-2/SIV_SM _viruses which accomplish nuclear localization by using accessory protein Vpx and G_2 _arrest by using Vpr [119]. Vpr/Vpx defective SIV virus, but not viruses defective in either protein alone, have been shown to have a greatly attenuated course with no progression to AIDS in rhesus monkeys, suggesting that both of these properties play significant roles *in vivo *[120]. Many studies also argue that nuclear localization rather than G_2 _arrest is important in macrophage infection of HIV-1. For example, HIV-1 transcripts in Vpr defective viruses lose the ability to be detected at some time between the reverse transcription and pro-viral DNA replication phases [41], suggesting that in the absence of Vpr the viral life cycle may be inhibited at the nuclear entry phase. The ability of IN to compensate for Vpr loss also suggests that nuclear localization plays a predominant role [14,44]. Therefore, there is ample evidence to support the notion that Vpr can induce nuclear localization independent of G_2 _arrest. Mutation studies have not demonstrated such independence, however, as the structure/function relationships have not proven separable.

As nuclear localization and G_2 _arrest seem to be related in some structural studies, it is not surprising that both properties of Vpr have been linked to productive infection of macrophages. Subbramanian and others argued that Vpr's ability to cause G_2 _arrest may also play a role in HIV-1 infection of macrophages [121]. Upon infecting macrophages with HIV-1 viruses that were Vpr WT, ATG-Vpr (Vpr negative), Vpr R62P (impaired in nuclear localization), and Vpr R80A (impaired in G_2 _arrest), the authors observed that unlike the Vpr R62P mutant, which only inhibited viral growth at low MOI, the Vpr R80A and ATG-Vpr viruses were the most impaired at higher MOI. However, R80A mutant, as expected, showed no differences as compared to the other mutants in the number of G_2 _stage cells in terminally differentiated macrophages, as these cells are already arrested. These results suggest that the so called G_2 _arrest property of Vpr is important in different ways than nuclear localization for productive viral infection in myeloid cells. While the authors hypothesized that the effect of G_2 _arrest on viral replication is due to biochemical properties of the mutant protein, the independence of these two properties in mutated Vpr constructs remains to be fully ascertained.

It is very important to note that the G_2 _arrest property of Vpr has been recently attributed binding to damaged DNA binding protein 1 and Cullin 4a-associated factor-1 (DCAF-1) [122-128] (originally identified as a binding partner called VprBP [129]), and is a result of subsequent induction of ataxia telangiectasia-mutated and Rad3 related (ATR) kinase. While it is unknown how Vpr/DCAF-1 binding promotes G_2 _arrest, it has been proposed that Vpr may recruit a particular factor to this complex, promoting ubiquitination and degradation of a yet unknown cellular protein or, perhaps, several targets [130,131]. Macrophages are non-dividing cells and are therefore not subject to the cell-cycle arrest function of Vpr and even lack the prerequisite ATR induction in the presence of Vpr [132]. The findings that demonstrate the importance of Vpr residues involved in G_2 _arrest in promoting HIV-1 replication likely suggest that the recruitment of native cellular factors to DCAF-1 promotes both properties. However, it is unknown what binding partners mediate these effects or if they are the same or overlapping for both G_2 _arrest and cellular permissiveness. A synopsis of these three properties and their effects on HIV-1 infection of macrophages is found in Figure 1.

The G_2 _arrest and HIV LTR promoter transactivation properties of Vpr may also be dependent or independent of each other. Many studies have shown that Vpr's ability to cause G_2 _arrest and increase viral production are linked [62,75,85,133,134]. While G_2 _cell cycle arrest may make HIV-1 infected T-cells and oddly macrophages, which are not dividing, more permissive to active infection, many studies have shown that Vpr constructs deficient in G_2 _arrest maintain the ability to function as a coactivator [59,84,90-92]. While G_2 _arrest and transactivation properties of Vpr both impart positive effects on viral replication, whether these effects represent independent functions is a matter of debate.

As mentioned previously, Vpr is believed to allow for permissive infection of HIV-1 in many cell types, but is considered particularly important for the infection of non-dividing cells such as macrophages and resting T-cells. As such, Vpr is likely important in generating a long lived reservoir for virus infection. Indeed, it has been suggested based on results in non-human primate studies, that macrophages are likely the main producers of virus in late stage simian/human immunodeficiency virus (SHIV) at a time when CD4^+ ^T-cells have been depleted [135]. In HIV-2/SIV_SM _virus, Vpr is hypothesized to have duplicated, giving rise to Vpx [5,136]. Vpr and Vpx have discrete functions in HIV-2/SIV_SM _viruses causing G_2 _arrest and nuclear localization respectively, whereas Vpr has both properties in HIV-1 [119]. Recently, it was shown that SIV/HIV-2 Vpx overcomes a block to reverse transcription in macrophages, further suggesting that HIV-1 Vpr may increase viral permissiveness in myeloid cells as well [137-139]. It is noteworthy to mention that Vpx also has such an effect on HIV-1 defective in Vpr, yet this effect is not seen with Vpr treatment. This likely suggests that Vpx acts on cellular targets that may be only partially in common to those of Vpr. Interestingly, Vpx binds DCAF-1 in a way similar to Vpr [125] and such interaction is necessary for the permissive effects described above. It has been suggested that Vpr and Vpx compete for binding to this complex and perhaps recruit unique or only partly overlapping binding partners [130]. Therefore, it is likely that the particular macrophage restriction factor antagonized by Vpx is not a target of Vpr. In agreement with this notion, previous studies have attributed Vpr to lifting a post-reverse transcriptional block, whereas Vpx seems to affect an earlier block in viral replication [41]. However, Vpr may use the same system to recruit other factors that promote permissive infection of HIV-1 into macrophages. It is unknown why HIV-1 Vpr does not possess the same properties as seen with Vpx in SIV or HIV-2, but obviously HIV-1 does not rely on these effects for successful infection *in vivo*. Considering that Vpr has small effects on macrophage permissiveness to HIV-1 during single a round of infection [140], but causes profound changes after long-term culture [40,41], it is likely Vpr mediated macrophage permissiveness has not been detected as compared to Vpx simply due to the a smaller magnitude of it's effect or due to short-term culture conditions.

HIV-1 virus is known to have anti-apoptotic properties in chronically infected macrophages and microglia [141], and causes a reduction of pro-apoptotic Bax expression in mitochondria of persistently infected cells [142]. While Vpr promotes apoptosis [143,144], it also exhibits anti-apoptotic properties [145]. It is noteworthy to mention that no study of which we are aware has ever shown toxicity of Vpr in macrophages. On the contrary, it has been argued that macrophages lack the ATR mediated the cell stress response normally induced by Vpr [132], which is required for the apoptotic activity that has been reported in other cell types. Intriguingly, Vpr was observed to inhibit apoptosis in a lymphoblastoid cell line by inducing Bcl-2, with concomitant downregulation of Bax in a manner seemingly contingent on Vpr expression level [145]. Further, Vpr mediates resistance to cell death from Fas ligand and TNF-α in these cells. The G_2 _arrest function of Vpr in these cells, however, is most likely defective since these clones exhibited cell cycle characteristics similar to those of control-transfected cells. As Vpr is toxic to non-myeloid cells, such as T-cells, the possible anti-apoptotic effects of Vpr that have been observed and attributed to Vpr in the study may be due to a low level of Vpr expression in the cell lines used. As such, the pro-survival effects of Vpr may need to be evaluated further. If Vpr promotes cell survival, it is conceivable that the pro-survival effects of HIV-1 may involve the action of Vpr, especially in macrophages, possibly in combination with additional host-viral interaction. In combination with the aforementioned abilities of Vpr to increase viral replication by inducing G_2 _arrest and activating the HIV-1 LTR, the potential of Vpr to promote infection of and survival of macrophages could be a highly significant factor in the development and/or maintenance of macrophage viral reservoirs. The differential mechanism of pro-apoptotic/anti-apoptotic Vpr activity warrants further investigation and may provide an avenue of therapy as an additive to highly active antiretroviral therapy (HAART), now renamed combination antiretroviral therapy (cART).

## Vpr and HIV dementia

**Figure 2:**
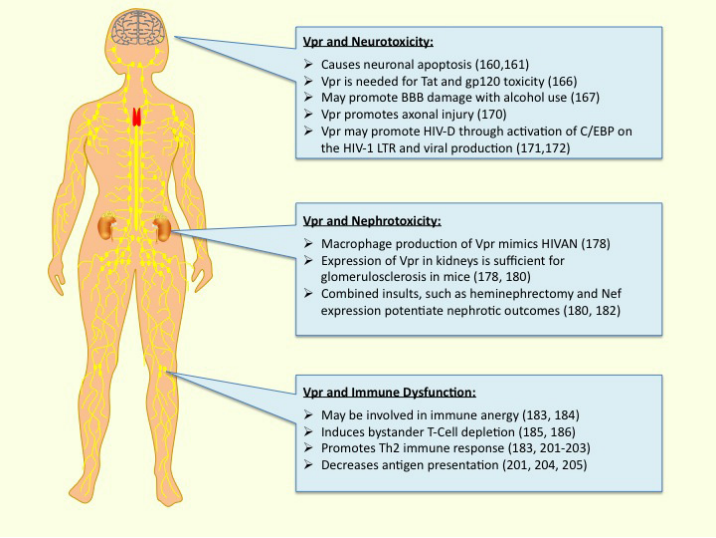
**Summary of HIV-1 pathology involving Vpr**. Vpr is likely important for both immune dysfunction as seen in AIDS and associated diseases including HIV-D and HIVAN.

HIV encephalopathy (HIV-E) is an associated underlying pathological condition seen in autopsy of patients with HIV-1 associated dementia (HIV-D), a disease characterized by motor and cognitive deficits. The presence HIV-1 virus in the brain is seemingly the cause of this condition as it was detected by *in situ *hybridization in patients with HIV-E but not in HIV-1 patents who do not exhibit this pathological condition [146]. Although the introduction of cART initially reduced the prevalence of HIV-D, the prevalence of HIV associated neurocognitive disorders (HAND) has been increasing (for review see [147]). While it is unclear if the minor and severe forms of HAND have common etiologic mechanisms, there is reason to suspect the importance of HIV infection in macrophages in the central nervous system (CNS) and/or the periphery, as well as the role of Vpr. Since Vpr has been implicated as both a direct and indirect contributor to the development of dementia, Vpr may also play a role in the more subtle forms of neurologic disease (Figure 2).

Although the principle mechanism of HIV-D pathology is not known, there is a preponderance of evidence suggesting that mononuclear cells play a critical role in disease progression. The major sources of HIV-1 production in the brain appear to be macrophages and microglia [146,148-150]. Furthermore, in brains of animals infected with SIV, perivascular macrophages are responsible for the majority of virus production, further implicating these cells in the pathology of CNS disease [151]. Macrophage/microglia numbers are more highly correlated with the severity of HIV-D than the presence of HIV in the CNS [152]. Patients with HIV-D also have elevated numbers of CD14^+^/CD16^+ ^monocytes in the periphery [153,154], which have neurotoxic properties *in vitro *[154]. CD14^+^/CD16^+^, HIV-1 positive macrophages have also been found in brains of patients suffering from HIV-D [155]. The presence of TNF-a protein and mRNA in patients with HIV-D has been reported to significantly correlate with the severity of symptoms in these patients, further suggesting that activated macrophage activity is directly involved in HIV-D pathology [152,156]. The increased number of CNS macrophages/microglia (in the absence of evidence for proliferation) suggests that the accumulation of myeloid cells in the brain is due to trafficking of peripherally derived macrophages [157], (reviewed in [158]). As mentioned previously, Vpr plays a significant role in the permissive infection of HIV-1 into macrophages and may increase the survival of infected myeloid cells; therefore, it is indirectly related to HIV-D pathogenesis.

Vpr may be a direct effector of HIV-1 mediated HIV-E pathology. Higher levels of Vpr have been found in the CSF of patients with HIV associated cognitive deficits. Vpr has been detected by immunofluorescence in the basal ganglia and frontal cortex of brains with HIV-E and is elevated in the serum and CSF of seropositive HIV patients [74,159] and has been shown to cause apoptosis *in vitro *[160]. The cells that contained Vpr in HIV-E brains were either macrophages or neurons. Transgenic mice that express Vpr in monocytoid cells display neuronal injury in the basal ganglia and subcortical area, which confirms *in vitro *findings [161]. Mechanistically, the neurotoxic effect of Vpr depends on the 70-96 C-terminal region, which is essential for the induction of neuronal apoptosis in striatal and cortical cells [162]. In neurons, this effect is mediated by activation of p53, caspase 9, and caspase 8 [161,163]. Although gp120 and Tat have also been shown to induce apoptosis in neuronal cells [164,165], intracellular Vpr expression in NT2 cells seemed to be necessary for the induction of apoptosis [166]. This effect many have even greater clinical relevance considering that Vpr and ethanol together cooperatively increase apoptosis in brain microvascular endothelial cells, which may possibly allow for greater blood brain barrier permeability to virus and infected cells [167]. Most recently, Vpr was shown to increase reactive oxygen species production in microglia and neuroblastoma cell lines, to lower ATP, to lower plasma membrane Ca^2+ ^ATPase (PMCA) protein levels, and increase cytoplasmic permeability in neuroblastoma cells [168]. By lowering PMCA levels, the efflux of Ca^2+ ^would be expected to increase in neuronal cells, which has been linked to cell death signaling in these cells (for review see [169]). Vpr produced from HIV-1 infected macrophages was found to impair axonal growth of neuronal precursors independently of apoptosis [170]. Vpr binds to CCAAT-enhancer binding protein (C/EBP) sites on the HIV-1 LTR [171] and consequently a C/EBP site with high affinity for Vpr, C/EBP I, is associated with clinical progression to HIV-D [172]. It has been proposed that Vpr activates C/EBP sites by direct binding to C/EBP I in the HIV-1 LTR, which has low affinity for C/EBP, as well as indirectly by upregulating the expression of C/EBP in host cells [173]. Vpr and Nef both induce RANTES/CCL5 chemokine in microglia, causing activation of brain mononuclear cells, which correlates with clinical dementia [174]. Therefore, Vpr is a direct and indirect mediator of cell death and neuronal impairment in HIV-1 patients as well as a necessary factor for the infection and survival of HIV infected macrophages, thereby further contributing to the pathogenesis of HIV-D.

## Vpr and HIVAN

HIV associated nephropathy (HIVAN) is a form of collapsing focal segmental glomerulosclerosis, largely due to HIV-1 protein toxicity to epithelial cells (for review see [175]). The most significant incidence of the disease is seen in HIV-1 positive patients of African descent, likely due to a prevalence of the MYH9 allele in this population [176]. As in HIV-D, macrophage trafficking and expression of virus has been implicated in pathology of HIVAN. Fibroblast growth factor 2 (FGF-2), which is elevated in kidneys of children with HIVAN, increases the attachment of uninfected and HIV-1 infected PBMC to tissue culture plates coated with renal tubular epithelium [177]. *In vivo*, FGF-2 likely increases the invasion of inflammatory cells into renal tissue, leading to renal injury. Interestingly, Vpr has been implicated in the development of HIVAN (Figure 2). A c-fms/Vpr transgene in mice produced focal glomerulosclerosis, suggesting that macrophage specific Vpr expression might be sufficient for kidney damage [178]. Further, it was reported that FVB/N mice expressing Vif, Vpr, Nef, Tat, and Rev in podocytes developed nephropathy and proteinuria suggesting that viral proteins themselves have toxic effects in the kidneys [179]. Vpr expressed in a transgenic mouse model demonstrated that presence of Vpr in podocytes is sufficient for glomerulosclerosis [180]. Lentiviral experiments *in vitro *produced similar findings [181]. Vpr expression in combination with Nef, however, results in severe kidney damage in transgenic mice [180]. Vpr expression combined with heminephrectomy also resulted in far more profound nephrotic changes [182]. The impact of heminephrectomy was almost entirely prevented by including treatment with angiotensin II type 1 (AT1R) receptor blocker olmesartan. To date, however, no specific therapies targeting Vpr/Nef nephrotoxicity or the attachment of affected macrophages to the tubular epithelium have been developed. It should be noted that in the studies using single or combined expression of viral proteins in particular cell types, such as in macrophages in the c-fms driven Vpr model, it is unclear if these effects occurred due to the secretion of these products from cells trafficking to the kidneys or due to other inflammatory cytokines produced in these cells due to the expression of these products.

## Vpr and immune dysfunction

Vpr has profound inhibitory effects on many members of the immune system involved in adaptive response (Figure 2). Consequently, Vpr reduces the efficacy of DNA and SIV-Nef vaccination *in vivo*, suggesting that Vpr may aid in evasion of immune response during HIV-1 [183,184]. The mechanism of immune dysfunction caused by Vpr appears to involve the induction of apoptosis and cell cycle arrest in bystander T-cells, contributing to the depletion of immune cells. While Vpr is seemingly anti-apoptotic in HIV-1 infected cell lines, *in vitro *studies suggest that bystander T-cells may be induced to undergo apoptosis in response to extracellular or secreted Vpr [145,185,186]. Although many studies argue that Vpr has effects outside of the infected cell due to secretion, this point remains controversial. However, *in vivo*, Vpr alone has been shown to be contribute to HIV-1 mediated immune dysfunction by promoting depletion of thymic cells (reviewed in [187]). Activation induced cell death by apoptosis has been proposed as a mechanism of HIV-1 infected CD4^+ ^lymphocyte depletion, although multiple mechanisms distinct from Vpr likely contribute to this process [188,189]. Vpr can increase Fas dependent caspase-8 dependent cleavage in T-cells to induce apoptosis, providing a potential mechanism for increased cell death. CD4 promoter-Vpr transgenic mice do show T-cell depletion in a Bcl-x, Bax, and caspase-1 dependent and Fas-Fas ligand independent manner [190]. G_2 _arrest precedes the induction of apoptosis by Vpr and has been reported to be necessary for progression to apoptosis [73], however, the latter findings remain controversial [191]. Recently, it was demonstrated that this property depends on Vpr activated phosphorylation of Chk1, an event that begins during the S phase of the cell cycle [192]. Apoptosis occurs via caspase-9 and seems to cause apoptosis in cancer cell lines with mutated p53, suggesting that this effect is independent of p53 function [193-195]. Vpr has also been postulated to increase the expression of TNF-α in dendritic cells (DC)s and in this way may indirectly promote apoptosis in CD8^+ ^T-cells [196]. The Vpr mediated depletion of uninfected T-cell populations likely contributes, in part, to the immune dysfunction observed in AIDS.

Recent studies have identified additional mechanisms of Vpr mediated T-Cell depletion. Vpr has been shown to upregulate natural killer group 2, member D (NKG2D) ligands in CD4^+ ^lymphocytes, which resulted in natural killer (NK) mediated toxicity to these cells [197,198]. It is unclear what effect Vpr has on HIV-1 infected CD4^+ ^T-cell depletion *in vivo*, since Vpr alone is sufficient to upregulate NKG2D ligand expression. Vpr could induce bystander T-cell killing due to NK mediated toxicity. It should also be mentioned, however, that Vpr has been reported to inhibit NK function [199,200], which would be predicted to oppose NK mediated toxicity. If infected T-cells are depleted due to NK function, this may suggest that the infection of these targets is outweighed by the advantage conveyed by immune suppression. Interestingly, the upregulation of NKG2 ligands by Vpr is also related to DCAF-1 binding in an ATR related mechanism, which suggests that these ligands may not be readily upregulated in macrophages that are reported to lack ATR response to Vpr expression [132,197,198]. Considering that macrophages have been reported to be the main viral reservoir during late stage infection of rhesus macaques with an SIV/HIV-1 chimeric virus (SHIV) [135], the depletion of T-cells may not be a limitation to virus persistence due to the availability of myeloid target cells. In summary, Vpr has been reported to cause apoptosis of bystander T-cells by multiple mechanisms, which may contribute to decreased immune function and possibly impaired viral clearance in the host.

Vpr may suppress cellular immunity by modulating antigen mediated activation and cytotoxic killing of surviving T-cells. *In vivo*, Vpr promotes a shift toward a Th2 response, likely by suppressing IFN-γ, a Th1 inducing cytokine [183]. Other studies have also confirmed that Vpr promotes Th2 cytokine IL-10 while suppressing the expression of Th1 cytokine IL-12 [201-203], presumably by modulating NF-κB response (discussed below). T-cell function also may be perturbed by downregulation of CD28 and CTLA-4 which are required for activation by antigen presenting cells and therefore adaptive immune function [204]. Recombinant Vpr has been shown to lower activation of macrophages and maturation of DCs by inhibiting the expression of key co-stimulatory molecules including CD40, CD80, CD83, and CD86 [201,205]. This suggests that Vpr may dampen antigen presentation by downregulation of partner molecules on both presenter and effector cells. Vpr has also been shown to suppress immune activation to superantigens *in vivo *[206]. More recently, Vpr has also been shown to modulate NK cell function, causing a reduction in cytolytic killing and differential regulation of IL-12 and TGF-β by Smad3 activation [200]. Therefore, Vpr may significantly contribute to the immune deficiency seen in AIDS by altering both adaptive and innate immune cellular function.

Evidence from many studies suggests that Vpr's effect on the immune system seems to be mediated by interaction with the NF-κB pathway by a mechanism involving GR. Glucocorticoids have been shown to have immunosuppressive effects due to NF-κB inhibition and induction of I kappa B alpha (IκBα), which prevents NF-κB translocation into the nucleus thereby preventing cytokine release and immune activation [207,208]. Vpr was first shown to induce T-cell apoptosis in a TCR dependent mechanism by inducing IκB and reducing NF-κB activity [209]. Vpr downregulates NF-κB inducible cytokines, including IL-2, IL-12, TNF-α, and IL-4, and chemokines, MIP-1α, MIP-1β, and RANTES [209-211]. These effects were reversed with RU486 treatment, suggesting that the inhibition of NF-κB via IκB induction mechanistically involves GR. Indeed, Vpr and GR cooperate to suppress NF-κB mediated transcription [95]. The cooperativity of Vpr with GR has been proposed as a cause of the hypersensitivity to glucocorticoids seen in HIV infected patients thus amplifying the GR induced immunosuppressive effect [210]. Recent studies, however, have reported that Vpr can increase NF-κB activity by inducing IκB phosphorylation and subsequent degradation [112]. Indeed, other studies have also shown that Vpr can induce NF-κB activity [212,213], therefore, the context in which these effects differ remains to be elucidated. Vpr's effects on the immune system seem to be carried out by several and possibly independent mechanisms. Therapeutic strategies targeting Vpr, therefore, may impair virus replication directly and at the same time serve promote functional antiviral immune responses.

## Targeting Vpr's effects as an adjuvant therapy to cART for HIV

**Figure 3:**
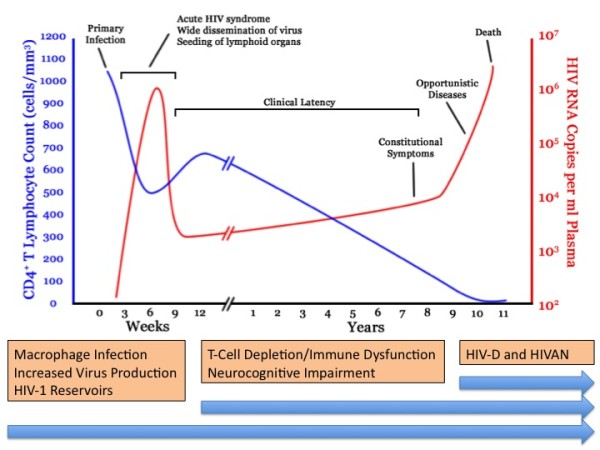
**Proposed timeline for HIV-1 Vpr mediated pathology and resistance to therapy**. Early in infection, Vpr allows for productive viral infection of macrophages. These cells contribute to virus production and drug resistant reservoirs seen throughout the infection. During clinical latency, Vpr contributes to the depletion of CD4^+ ^and CD8^+ ^T-cells, as well as interferes with antigen presentation. Such properties may contribute to HIV-1 escape from immune surveillance, and effective humoral control of HIV infection. While it is yet unclear if neurocognitive dysfunction and HIV-D are related pathologies, Vpr mediated immune dysregulation and neurotoxicity may contribute to early neurological impairment in HIV-1 patients. Late in HIV-1 pathogenesis, increased expression of viral proteins including Vpr, contributes to the development of associated pathologies, such as HIV-D and HIVAN.

The actions of Vpr in the virus life cycle and its role in the pathogenesis of HIV induced immune dysfunction and end-stage organ disease suggest the potential importance of Vpr as a therapeutic target for the treatment of HIV infection (Figure 3). Several additional key observations have provided additional support for this notion. Vpr/Vpx defective SIV virus has been shown to have a greatly attenuated course with no progression to AIDS in rhesus monkeys [120]. In HIV-1, Vpx is absent and Vpr is thought to carry out Vpx functions, suggesting that in humans a Vpr deletion would have similar effects. Infection of Vpr defective HIV-1 into tonsilar histocultures showed a fifty percent reduction in HIV-1 production, even though macrophages represented a small portion of total infectable cells [214]. Further, an accidental infection of a lab worker with HIV-1 containing a frame shift mutation in codon 73 of the Vpr gene as well as infection of rhesus macaques with Vpr mutated virus resulted in spontaneous reversion of the Vpr defective virus to the WT phenotype, which implies that Vpr containing virus obtained a selective advantage over the Vpr mutant [134,215]. Vpr has also been shown to reduce the efficacy of DNA and SIV-Nef vaccination *in vivo*, suggesting that in the absence of Vpr a more effective immune response to HIV would be possible [183,184]. Finally, a recent study of six vertically infected children that presented as long-term nonprogressors reported that every patient had a mutated Vpr gene in addition to mutations in other genes that were not present in all patients [216]. Interestingly, all of these mutations involved a decrease in Vpr's apoptotic effects, suggesting that the cytotoxic properties of Vpr are of key clinical importance. However, another report suggests that these effects are more related to nuclear localization [217]. One of the major clinical consequences of Vpr in HIV-1 infected patients is the existence of viral reservoirs in macrophages. Nucleoside reverse transcriptase inhibitors (NRTIs) are more effective in macrophages than in CD4^+ ^T-cells for early viral inhibition; non-NRTIs are equally effective in macrophages and in CD4^+ ^T-cells for early infection (for review see [218]). Protease inhibitors, however, require a much higher dose to effectively control HIV-1 infection in macrophages than in CD4^+ ^T-cells, and it is unknown if they achieve the concentrations needed to inhibit macrophage mediated HIV-1 production in compartments such as CNS or testes. While NRTIs, non-NRTIs and protease inhibitors prevent the cell to cell spread of HIV-1 infection, it is unknown how efficiently these drugs address virus produced from infected macrophages *in vivo*. There is currently no therapeutic approach for eliminating macrophage reservoirs that represent drug resistant reservoirs of HIV-1 infection and contribute to the pathogenesis of AIDS. Nanotechnology-based drug delivery systems have been proposed as one method for delivering drugs more effectively to macrophages, especially those in relatively inaccessible body compartments [219,220]. These novel technologies offer ways to better deliver currently available medications, but do not address the survival of persistently infected HIV-1 reservoirs.

A therapeutic approach to target HIV-1 infected mononuclear cells would be to employ specific cytokines or cellular kinase inhibitors. One candidate, TNF-related apoptosis-inducing ligand (TRAIL), has been shown to cause HIV-1 infected macrophages to undergo cell death. However, M-CSF, which is upregulated in HIV-1 infected cells, downregulates TRAIL-R1/DR4 [221]. Imatinib, a tyrosine kinase inhibitor that has some cross reactivity to colony stimulating factor-1 receptor (CSF-1R), the receptor for M-CSF, restores the effect of TRAIL on infected MDM cells [221]. TRAIL has been shown to act through the PI3/Akt pathway [222] and consequently other PI3/Akt inhibitors have similar effects on infected MDM cells [223]. Additionally, morphine in combination with gp160 has been shown to cause apoptosis in mononuclear cells [224]. In combination with cART therapy, a clinical approach to target the anti-apoptotic pathways in HIV-1 infected macrophages may yield more effective therapies.

Another approach for targeting macrophage reservoirs is to target the specific host mediators of Vpr function. Heat shock proteins have been proposed as cellular targets of Vpr and a mechanism of antiviral response (for review see [225]). HSP 27 inhibits Vpr dependent G_2 _arrest and cell death in T-lymphocytes when expressed exogenously, but does not seem to inhibit viral replication in macrophages [226]. Another heat shock protein, HSP 70, can inhibit HIV-1 replication in a Vpr dependent manner as well as reduce G_2 _arrest in proliferating cells [227]. HSP 70, however, can replace Vpr function in Vpr defective viruses as well as have anti-viral properties in non-proliferating macrophages [228]. As heat shock response is protective, increasing heat shock pathways could promote the survival of chronically infected cells. In light of the recent findings suggesting that Vpr mediated apoptotic effects are important in pathogenesis, and that G_2 _arrest apoptosis and NK mediated destruction of T-cells depends on Vpr binding to DCAF-1, targeting the Vpr ubiquitination pathways may also be useful for clinical intervention. Additionally, HAX-1 associates with Vpr, and suppresses Vpr pro-apoptotic effects, suggesting that molecules that bind to this site on Vpr may be used to neutralize Vpr's immunosuppressive effects [229]. Alternatively, the anti-apoptotic effects of Vpr in HIV-1 infected cells may contribute to the persistence of viral reservoirs *in vivo*. The Tat mediated upregulation of c-Flip, which prevents TRAIL toxicity, has been proposed as one mechanism of the differential effects of Vpr in infected and non-infected cells and may prove to be a good target for inducing apoptosis in chronically HIV-1 infected macrophages [230].

Several pharmacological approaches have already been suggested to target Vpr pathways. As many Vpr mediated effects depend on GR activity, RU486 has been proposed as a therapy for HIV-1 and has been shown to suppress HIV-1 replication in infected mononuclear cells and to suppress Vpr mediated downregulation of IL-12 and other cytokines [117,209]. Vpr is necessary for viral PIC entry into the nucleus of non-dividing cells and therefore this property of Vpr has also been investigated as a potential avenue of therapy. CNI-H0294, a specific inhibitor of HIV nuclear localization, was shown to indeed inhibit viral production. It was found to diminish infection in PBMCs and macrophages, which would not necessarily deplete viral reservoirs but may help prevent new macrophage infection [231]. More recently, a study has demonstrated that hematoxylin is a specific inhibitor of the Vpr/importin-α interaction and consequently prevented the nuclear import of the HIV PIC complex [232]. In summary, many studies have proposed targeting the cellular effects of Vpr as a way of treating the consequences of Vpr function in HIV-1 infection. In combination with established cART regiments, these approaches may lower viral loads, increase immune response, and even contribute to the depletion of viral reservoirs thus improving the clinical outcome in HIV patients.

## Vpr as a pharmacotherapeutic and delivery agent

Vpr is a multifunctional protein that is able to efficiently facilitate many HIV-1 functions. Some of these properties, however, lend themselves for use in the clinic. Importantly, Vpr can traffic into cells [75] and is incorporated into HIV particles [12,233]. Further, the Vpr peptide region from R14-88 has been used to introduce other protein products into HIV-1 particles [234]. As a result, Vpr has been explored as a vector system for drug delivery by conjugation to apolipoprotein B mRNA editing enzyme, catalytic peptide 3G (Vpr14-88-Apobec3G) [235]. Apobec3G has strong antiviral effects in Vif deficient viruses, but in the presence of Vif loses the ability to incorporate into virons and therefore its therapeutic efficacy [236,237]. The fusion of Vpr 14-88 to Apobec3G facilitates packaging into the HIV-1 particles and restores the ability of Apobec3G to inhibit viral replication. These studies demonstrate that the use of Vpr to amplify the effect of antiviral drugs or facilitate drug delivery is a promising avenue for HIV therapy.

The discoveries of other properties of Vpr, including induction of G_2 _cell cycle arrest and apoptosis, have led the argument that Vpr has efficacy as an anti-cancer agent [238]. Further, Vpr induction of apoptosis seems to be independent of p53 function, suggesting that mutations in p53 commonly seen in various tumor types will not prevent the potential therapeutic efficacy of Vpr [193]. Other studies have also provided support for the anti-cancer application of Vpr by showing that Vpr induces greater apoptosis in cells undergoing active replication, implying that this toxic effect would be particularly targeted to cancer cells [73,209]. However, Vpr, like other chemotherapeutic agents, also possesses the ability to transform cells as double stranded breaks and aneuploidy have been reported in cell lines [239]. The consequences of Vpr on mitogenic transformation *in vivo *require further assessment and remain one potential limitation of such a therapeutic approach.

## Conclusion

More than two decades of research on Vpr has greatly contributed to the knowledge scientists and clinicians have available about HIV-1 pathogenesis. The findings revealed that Vpr, while not essential for viral replication per se, is a biologically important, playing a critical role in the infection of non-dividing target cells including macrophages and resting T-cells. Vpr promotes infection of dividing as well as non-dividing cells through a variety of effects including, nuclear localization, cell cycle arrest, apoptosis, and other effects due to DCAF-1 binding, as well as transactivation of host and viral genes. These activities of Vpr are likely responsible for many aspects of HIV-1 infection as well as associated pathology seen in AIDS. With the advent and success of cART therapy, HIV-1 infection has transformed from an untreatable disease to a more manageable chronic condition. Current investigations for new therapies represent an ongoing area of basic science research that holds great priority due to cART resistant reservoirs of HIV-1 infection *in vivo*. Vpr mediated pathogenesis is one avenue of investigation that holds promise when combined with other therapeutic approaches. Further basic and translational studies will be required to generate future therapeutic advances targeting Vpr function. Such studies could target Vpr as well as a variety of host-virus interaction pathways.

## Competing interests

The authors declare that they have no competing interests.

## Authors' contributions

MK conducted a literature review and wrote the above material. MK also created the figures presented in the paper. JR organized the ideas presented in this review and helped to edit the content so that it is relevant to current HIV research. JR also suggested ideas on how to illustrate the figures and highlighted important concepts that needed to be included. Both authors read and approved the final manuscript.
